# Altered reward and effort processing in children with maltreatment experience: a potential indicator of mental health vulnerability

**DOI:** 10.1038/s41386-022-01284-7

**Published:** 2022-02-11

**Authors:** Diana J. N. Armbruster-Genç, Vincent Valton, Louise Neil, Vivien Vuong, Ze Freeman, Katy C. Packer, Marianne J. Kiffin, Jonathan P. Roiser, Essi Viding, Eamon McCrory

**Affiliations:** 1 Division of Psychology and Language Sciences, University College London, London, UK; 2 Institute of Cognitive Neuroscience, University College London, London, UK

**Keywords:** Reward, Stress and resilience, Risk factors

## Abstract

In this longitudinal study of children and adolescents with a documented history of maltreatment, we investigated the impact of maltreatment on behavioral and neural indices of effort-based decision making for reward and examined their associations with future internalizing symptoms. Thirty-seven children with a documented history of maltreatment (MT group) and a carefully matched group of 33 non-maltreated children (NMT group) aged 10–16, completed an effort-based decision-making task during functional magnetic resonance imaging (fMRI). Internalizing symptoms were assessed at baseline and again 18 months later. Computational models were implemented to extract individual estimates of reward and effort sensitivity, and neural signals during decision-making about different levels of reward and effort were analyzed. These were used to predict internalizing symptoms at follow-up. We identified lower effort-related activation in the anterior cingulate cortex (ACC), a prespecified region-of-interest, in the MT relative to the NMT group. No group differences were observed in the striatum, or in behavioral indices of reward and effort processing. Lower effort-related ACC activation significantly predicted elevated internalizing symptoms at follow-up in the MT group. These findings suggest that disrupted effort-related activation may index latent vulnerability to mental illness in children who have experienced maltreatment.

## Introduction

Maltreatment experienced during childhood, such as neglect, physical, emotional, and sexual abuse is associated with a higher risk of poor mental health across a broad range of psychiatric disorders [1]. While these relationships are well established, there is still limited understanding of the mechanisms underlying them. At the neurocognitive level, it has been proposed that certain brain systems adapt in ways that may confer immediate benefits in atypical environments associated with maltreatment, but which are poorly optimized for more normative environments [2]. This may, over time, contribute to what has been conceptualized as “latent vulnerability” for a psychiatric disorder [2]. Candidate cognitive processes in which such changes have been proposed to confer latent vulnerability include threat processing [3], autobiographical memory processing [4], executive control [5], affect regulation [6], and reward processing [7, 8].

As negative outcomes following maltreatment are extremely diverse, it is likely to be most fruitful to focus on putative transdiagnostic mechanisms implicated in multiple disorders [9–11]. A range of common mental health disorders associated with the experience of maltreatment—including depression, disruptive behavior disorders, and addiction—are characterized by atypical reward processing at both behavioral and neural levels [11–13]. Reward processing has received particular attention in the field of depression (for a review see [13]), and has also been implicated in anxiety (e.g., [14]). Converging evidence shows blunted reward anticipation and poorer ability to adapt behavior as a function of reward in depression, as well as lower neural activation in fronto-striatal circuitry [15], including the striatum and anterior cingulate cortex (ACC) [16, 17]. Importantly, these alterations are more than simply correlates of concurrent illness, as they have been shown to predict the subsequent onset of symptoms in longitudinal work [18]. This suggests that altered reward processing is mechanistically implicated in the development of depression.

Developmental histories of abuse and neglect can be characterized as representing atypical experience of reinforcement. In such early adverse environments, rewards and punishments are less predictable and may be qualitatively different from those encountered during more normative rearing experiences. For example, they may be more skewed towards punishment and absence of reward [19, 20]. Several studies of children and adults who have experienced childhood maltreatment report altered behavioral responses towards reward [21, 22], increased apathy [23, 24], and blunted neural responses, specifically within fronto-striatal circuitry, during reward anticipation [7, 21, 25–27], or during the viewing of positive stimuli [8], relative to individuals with no history of maltreatment. One region frequently implicated across studies is the striatum [7, 21, 27], a key area for detecting and anticipating rewards [28]. Research using animal models of early-life adversity suggests that neurotransmission in this reward circuit based on the corticotropin-releasing factor might be disrupted leading to aberrant development of neural circuits processing reward signals [29].

While this research has begun to broaden our understanding of the effects of early adversity on reward processing, extant studies have focused on a limited set of reward-related processes, specifically reward anticipation and consummation. This contrasts with the more fine-grained investigation of reward processing implicated in specific psychological processes underpinning motivation, and the use of sensitive computational approaches that have been adopted in recent studies of both healthy [30] and depressed individuals [31, 32]. Delineating the way in which reward and effort processing is altered following maltreatment experience is essential if we are to develop more precise mechanistic models of psychiatric vulnerability. Husain and Roiser [33], for example, have proposed a framework within which effort-based decision-making for rewards can be experimentally investigated, particularly in the context of depression, a disorder commonly associated with maltreatment experience [34].

In one study investigating apathy in a preclinical healthy sample, Bonnelle et al. [35] developed a computational effort-based decision-making task for rewards providing individual measures of effort discounting and reward sensitivity. This task has good ecological validity as participants are required to exert real physical effort to gain rewards. In this non-clinical sample, individuals reporting higher levels of apathy were less willing to exert effort when rewards were small. A subsequent functional magnetic resonance imaging (fMRI) study using the same task reported that apathy scores were associated with lower effort-related activation in a subset of the effort-processing network, namely in the supplemental motor area (SMA) and cingulate motor zones [36]. This is in line with extant prior research which robustly implicates the ACC as a major hub for the processing of reward for effort both from human (e.g., [37–42]) as well as animal studies (e.g., [43, 44]).

However, despite considerable interest in understanding reward processing in children with maltreatment experience, there are no studies to date investigating effort-based decision making for reward in this population. Furthermore, with two exceptions [7, 27], studies of reward processing in maltreated individuals have been cross-sectional in design. A longitudinal design is necessary to shed light on whether any observed differences associated with maltreatment experience reflect a latent vulnerability for internalizing psychopathology.

The aim of the present study was to systematically investigate the behavioral and neural underpinnings of effort-based decision making for reward and their predictive value for future internalizing symptoms assessed at an 18-month follow-up, in children and adolescents with a documented history of maltreatment. Using an adapted version of the task developed by Bonnelle and colleagues [36] during fMRI, we were able to assess the propensity to exert effort for reward in an engaging task that involved actual physical effort. We employed a Bayesian modeling approach to extract precise individual measures of reward and effort sensitivity and examined neural responses to different levels of reward and effort in a parametric fashion.

The theory of latent vulnerability posits that less frequent rewards and unpredictable punishments may lead to neurocognitive alterations in reward processing which, over time, increase vulnerability to internalizing symptomatology. We predicted that, during effort-based decision making for reward, those with maltreatment experience compared with non-maltreated peers, would present with: (i) attenuated brain activation with increasing effort levels in the striatum and ACC; and (ii) attenuated reward-related activation in the striatum. We based these predictions on prior neuroimaging studies of reward in individuals with maltreatment experience [21, 26, 27] and computational studies of effort and reward in non-maltreated adult samples [36, 41, 45, 46]. Furthermore, we predicted that any observed group differences in the neural response in the striatum or ACC would be associated with increased risk for future internalizing symptoms at follow-up. This hypothesis was based on the relationship between attenuated reward processing and depression symptoms found in samples with maltreatment experience [7, 27]. At the behavioral level we predicted greater effort sensitivity in those with maltreatment experience [35, 36], however, we note that previous research did not find consistent differences on the behavioral level [7, 21, 22].

## Method

### Procedure

At baseline, participants completed an fMRI session and along with carers completed a set of questionnaires. Follow-up data was collected for most participants during a phone interview or in an internet-based questionnaire due to the Covid-19 pandemic. The follow-up interval was approximately 1.6 years ± 1 month, with no significant difference between the groups (*t*(56) = −0.72, *p* = 0.475; Table S4).

### Participants

The study was approved by the UCL Research Ethics Committee. A total of 39 young people aged 10–16 years with a documented history of maltreatment (maltreatment group, MT) were recruited from Social Services London. A sample of 37 typically developing young people (non-maltreatment group, NMT) was recruited via London schools and community adverts to match the maltreatment group with regards to sex, age, pubertal status, ethnicity, socioeconomic status (SES), and IQ (Table 1). Consent was provided by parents, carers, or social workers if legal guardianship was with local authorities. All young people provided their assent for participation. Exclusion criteria included pervasive developmental disorder including autism, neurological disorders, IQ < 70, and standard MRI contraindications. Two participants from the MT group and four from the NMT group had excessive head movement and were excluded from further analyses (SI; Table S1) leaving a total of 37 MT and 33 NMT participants. The behavior for one MT participant could not be fitted with the computational models and was excluded from these analyses. A total of 27 MT and 31 NMT participants completed the follow-up.

**Table 1:** Background and psychopathology measures (for more information on all recruited participants see Table S1).

	Baseline			Follow-up		
	MT Group (*N* = 37)	NMT Group (*N* = 33)	*p*	MT Group (*N* = 27)	NMT Group (*N* = 31)	*p*
**Background Measures**						
Sex, female: *n* (%)	21 (57)	21 (64)	0.558	17 (63)	19 (61)	0.896
Age (*SD*)	13.8 (2.0)	13.3 (2.2)	0.367	15.2 (1.9)	15.0 (2.2)	0.720
Pubertal status (*SD*)	2.7 (0.8)	2.4 (0.9)	0.138	2.8 (0.9)	3.0 (0.8)	0.543
Ethnicity, White: *n* (%)^a^	15 (41)	13 (39)	0.922	9 (33)	13 (42)	0.501
Socioeconomic status (*SD*)	3.3 (1.0)	3.2 (0.8)	0.689	3.0 (1.0)	3.2 (0.7)	0.315
Wechsler Abbreviated Scales of Intelligence II IQ (*SD*)	100.7 (11.7)	103.6 (9.6)	0.260	101.7 (11.1)	103.3 (9.8)	0.574
**Psychopathology measures**						
SDQ total score (*SD*) (parent report)	11.0 (6.6)	6.7 (5.2)	0.004*	11.6 (6.9)	8.7 (5.2)	0.085
Emotional symptoms (*SD*)	2.7 (2.2)	1.7 (1.9)	0.046	3.8 (2.7)	2.2 (2.1)	0.013*
Conduct problems (*SD*)	2.4 (2.4)	1.1 (1.6)	0.007^b^	1.8 (2.0)	1.5 (1.4)	0.473^b^
Hyperactivity (*SD*)	4.0 (2.6)	2.2 (2.0)	0.002^b^	3.7 (2.5)	2.7 (2.2)	0.093^b^
Peer problems (*SD*)	1.9 (1.5)	1.8 (1.8)	0.897^b^	2.3 (1.7)	2.4 (1.9)	0.769^b^
Prosocial behavior (*SD*)	8.2 (1.8)	9.1 (1.4)	0.040^b^	8.3 (2.0)	8.3 (1.5)	0.988^b^
CASI major depressive episode (*SD*) (parent report)	6.4 (4.4)	4.3 (4.7)	0.060^b^	5.6 (4.0)	5.4 (4.7)	0.800^b^
CTQ (*SD*) (child report)	18.1 (10.7)	12.9 (3.2)	0.020^b^	–	–	–

*SDQ* Strengths and Difficulties Questionnaire; *CASI* Child and Adolescent Symptom Inventory; *CTQ* Childhood Trauma Questionnaire.

*p < 0.05 surviving correction for multiple comparison

^a^For a complete breakdown of ethnicities see Table S3.

^b^for illustrative purposes only, not part of hypothesizing/interpretation.

### Measures

#### Maltreatment history

All participants in the maltreatment group had experienced a level of maltreatment requiring the intervention of social services. Occurrence and severity of neglect, emotional abuse, sexual abuse, and home violence including physical abuse and/or intimate partner violence were rated by the young person’s social worker using social services’ file information with ratings ranging from zero (not present) to four (severe) [47] (SI; Table S2).

#### Background measures

Cognitive ability was assessed at baseline using two subtests of the Wechsler Abbreviated Scales of Intelligence (WASI-II) [48]. Pubertal status was assessed by both parent and child report using the Pubertal Development Scale (PDS) [49]. SES was measured as the carer’s educational level ranging from one (postgraduate degree) to five (no formal education).

#### Psychopathology

Parents/carers completed the Strengths and Difficulties Questionnaire (SDQ) [50] assessing general functioning and the Child and Adolescent Symptom Inventory (CASI) [51] assessing depressive symptomatology and other symptom areas both at baseline and follow-up. Young participants completed the Childhood Trauma Questionnaire (CTQ) [52] at baseline. At follow-up, all carers completed the Coddington Life Events Scale with appropriate child (up to the age of 12) and adolescent (13 and older) versions [53]. For those participants who were followed up after the start of the Covid-19 pandemic, carers also completed the Coronavirus Health Impact Survey (CRISIS) ‘Baseline Current Form’ (https://github.com/nimh-comppsych/CRISIS) assessing the impact of the pandemic on the child from which an emotional impact subscale was calculated (SI).

### Apparatus and Task

**Fig. 1:**
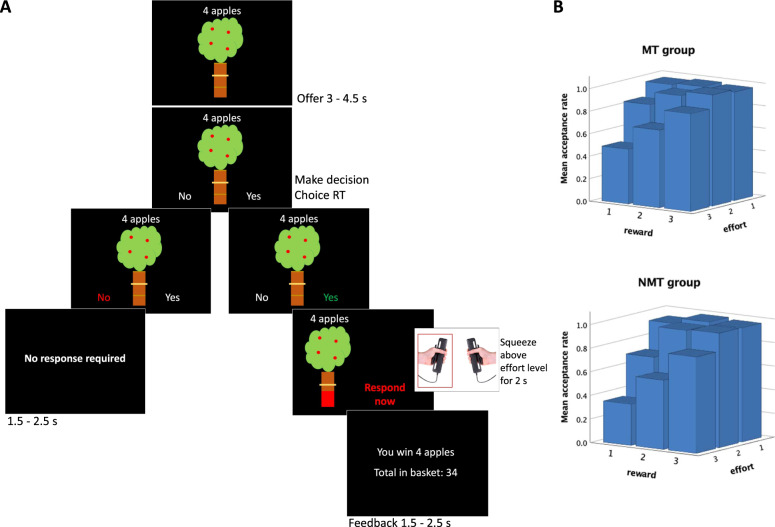
Effort-based decision making for reward task and behavioral descriptives. **A** On each trial, participants were presented with an offer consisting of a given reward (4, 8, or 12 apples) for a given effort (20%, 50%, or 80% of their maximum voluntary contraction) presented as apples on a tree with a yellow bar on the trunk indicating the effort level. They indicated their decision by applying a mild force to either the left- or right-hand dynamometer. If they decided to accept the offer, the tree reappeared on either the left or the right side of the screen indicating which hand dynamometer the participant had to use to apply the required effort level, which they had to sustain for at least 2 s over a 4 s period. Depending on whether the participant was successful or not they received feedback; points were never deducted. **B** Mean acceptance rates by reward and effort for MT and NMT group.

Task stimuli were presented in Matlab (Mathworks Inc.) using Psychtoolbox (http://psychtoolbox.org). Force data were acquired using two (one for each hand) TSD121B-MRI hand dynamometers (BIOPAC Systems Inc.) which were calibrated individually for each participant. Prior to entering the scanner, participants were instructed and familiarized with the task and use of the hand dynamometers. During training, head movement was fed back to the participants to reduce any head movements. The task was adapted from Bonnelle and colleagues [36] (Fig. 1A) including three reward and three effort levels, resulting in a reduced number of trials to make it better tolerated by children. Participants completed a total of 72 trials over three runs lasting approximately six min each with short breaks in between. The instructions included that there were no right or wrong answers but that participants should rather ask themselves, whether the reward was worth the effort. Participants were also instructed that they would receive a voucher depending on how many points they won in the game.

### Computational analyses

A variety of computational models with increasing complexity were built to capture the influence of reward and effort levels on decisions to exert effort. Model comparison was performed to select the most parsimonious model (i.e., the model that best captured the participants’ performance, whilst penalizing for model complexity). This allowed us to test various hypotheses of how reward and effort contributed to decisions, as well as estimating parameters for each participant corresponding to reward and effort sensitivity.

All models were implemented in a hierarchical logistic regression framework, such that we could model trial-by-trial decisions based on the effort and reward on offer on a given trial. Parameter estimates were recovered using hierarchical Bayesian estimation, which is more accurate than standard maximum likelihood methods, through the application of soft constraints on likely parameter ranges using prior distributions [54]. For brevity, we only present the winning model in the main text (further details in SI).

The winning model comprised five parameters per participant: two effort sensitivity parameters, a linear and a quadratic term—the latter captures disproportionate changes in the propensity to accept offers at higher effort levels—yielding a unique subjective perceived effort profile for each participant; a linear reward sensitivity term, reflecting subjective perceived reward; an intercept term capturing the general propensity to accept offers, independent of effort and reward levels; and a noise term capturing randomness in responding due to inattention or mistakes (see SI). The parameters were then compared between the groups using *t*-tests in SPSS25.

Where applicable, multiple comparisons of non-independent tests were corrected using the Benjamini–Hochberg procedure.

### fMRI analyses

Data were analyzed using SPM12 (https://www.fil.ion.ucl.ac.uk/spm/software/spm12) implemented in Matlab R2017a (MathWorks Inc.).

Subject-level analysis: Each individual’s preprocessed data (see SI) was modeled using a general linear model. Task-related activation was assessed through the inclusion of regressors corresponding to each condition of interest, time-locked to the relevant events, and convolved with SPM’s synthetic hemodynamic response function: (1) combined decision/choice phase (jittered offer presentation plus response period, 3–4.5 s), parametrically modulated on each trial by (i) subjectively perceived reward, (ii) subjectively perceived effort [(i) and ii) were calculated using each participant’s computationally derived estimates for subjective reward/effort sensitivity] and (iii) cost-benefit weighing (defined as the absolute value of the acceptance rate minus 0.5, as previously described [36]); (2) exertion of physical effort (4 s), parametrically modulated by the average strength applied on each trial; (3) outcome (1.5–2.5 s jittered) parametrically modulated by the reward obtained (see SI for further details on fMRI methods and results of models including actual reward and effort levels, Table S10).

Region-of-interest (ROI) analyses: To test our primary hypotheses regarding the striatum and ACC, ROIs were identified through the Neurosynth database (www.neurosynth.org) using the keyword ‘effort’. As the ACC is a functionally diverse brain area and in absence of a comparable study on a developmental sample, this approach was considered the most objective way to localize our regions of interest and is in line with prior neuroimaging studies of adolescents with childhood maltreatment experience [27]. Peak voxels were extracted and used to build spherical ROIs with 6 mm radius (Table 2).

**Table 2:** ROI coordinates and results of group comparisons for reward- and effort-related activation (bilateral structures were combined).

Regions of Interest							
				Comparison for reward-related activity		Comparison for effort-related activity	
	Coordinates			*t*	*p*	*t*	*p*
	x	y	z				
ACC	0	+14	+46	−0.54	0.588	2.19	0.032*
Right striatum	+22	+14	+6	0.41	0.686	0.32	0.751
Left striatum	−14	+6	+10				

*p < 0.05.

Second-level analysis: Contrast estimates from ROIs were extracted from the parametric subjective reward and parametric subjective effort modulators on the decision/choice phase and subjected to *t*-tests using SPSS25.

Exploratory whole-brain analyses (*p* < 0.05 FWE cluster corrected; for details see SI), were employed to explore group differences in both reward and effort processing during the decision/choice phase outside the pre-specified ROIs. Covariates of no interest (age, sex, and pubertal status) were included in all second-level analyses.

### Longitudinal analyses

A linear regression model was computed predicting emotional symptoms in the MT group at follow-up with baseline emotional symptoms and ACC effort-related activity (as this was where group differences were detected, see Results) entered as regressors, as well as sex, age, pubertal status, and MT severity.

### Power analysis for observed data

Using G*Power [55] we calculated that, given our sample size, we had at least 80% power to detect group differences of *d* > 0.75 at alpha = 0.05 (two-tailed), comparable to effect sizes reported in previous cross-sectional [25] and longitudinal work [7].

## Results

### Psychopathology and longitudinal changes

The MT group, compared to the NMT group, reported higher levels of internalizing symptoms as indexed by the SDQ Emotional Symptoms at baseline and follow-up and general psychopathology as indexed by the SDQ total score at baseline (Table 1). In the MT group there was also a significant increase of emotional symptoms between baseline and follow-up (mean at follow-up = 3.8, SD = 2.7; *t*(26) = 2.37, *p* = 0.025). Depression symptoms, as indexed by the CASI, were marginally higher at baseline in the MT group but did not differ between groups at follow-up (Table 1). In our subsequent analyses, we, therefore, focus on the SDQ Total Score (indexing overall psychological functioning) and SDQ Emotional Symptoms (indexing internalizing symptoms).

Among those for whom the pandemic began in the follow-up period, groups did not differ on reports of its emotional impact (*t*(13.2) = −0.50, *p* = 0.629). Number of negative life events during the follow-up period also did not differ significantly between groups (χ^2^(1, N = 58) = 4.0, *p* = 0.135 respectively; see also SI, Table S4).

### Analysis of behavior

Figure 1B shows the mean acceptance rates by reward and effort for both groups separately. An ANOVA on acceptance rate with the factors reward, effort and group showed expected main effects of reward (*F*(1.31,87.8) = 51.4, *p* < 0.001; partial η^2^ = 0.43) and effort (*F*(1.24,82.8) = 94.1, *p* < 0.001; partial η^2^ = 0.58) as well as their interaction (*F*(2.61,174.9) = 27.5, *p* < 0.001; partial η^2^ = 0.29); MT participants accepted offers more often overall than NMT participants, however this did not survive correction for multiple comparisons. All interactions with group were non-significant. There were no significant group effects for response time or for success rates (SI; Table S5 and S6, Fig. S3), and the groups did not differ significantly on total rewards collected (*t*(68) = −0.69, *p* = 0.491).

Analyses of computational parameters did not detect any significant differences between the groups in overall propensity to accept offers (intercept parameter: *t*(67) = −1.46, *p* = 0.150), sensitivity to increasing reward (linear reward parameter: *t*(67) = −0.38, *p* = 0.706), sensitivity to increasing effort (linear effort parameter: *t*(67) = 1.18, *p* = 0.244; quadratic effort parameter: *t*(67) = −0.733, *p* = 0.466), or random responding (noise parameter: *t*(67) = −0.143, *p* = 0.887).

In exploratory analyses, correlation analyses were conducted with our overall measure of psychopathology (SDQ Total Score) and measure of internalizing symptoms (SDQ Emotional Symptoms). A negative correlation was found between reward sensitivity and SDQ Total Score in the MT group although this did not survive correction for multiple comparisons (SI Table S7).

### fMRI: ROI analyses

**Fig. 2:**
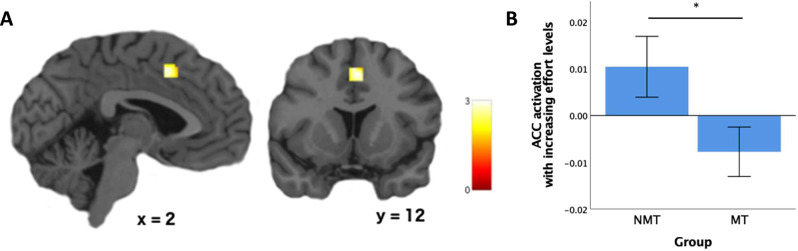
ACC ROI location and activation levels. **A** ACC ROI showing decreased activation with increasing effort levels in the MT group compared to the NMT group (pre-specified ACC ROI mask applied to two-sample *t*-test map in SPM). **B** Group difference in average ACC ROI activation with increasing effort levels during the decision/choice phase (error bars +/− 1 SE). *p < 0.05.

Table 2 shows the locations of ROIs (derived from Neurosynth) and the p-values for group comparisons (bilateral structures were combined). Since we had specific a priori hypotheses regarding the ACC and striatum based on existing literature, we did not apply correction for multiple comparisons to these analyses. Parametric activation with increasing effort levels in the ACC ROI was significantly higher in the NMT than the MT group (*t*(67) = 2.19, *p* = 0.032; Cohen’s *d* = 0.52) (Fig. 2).

An exploratory analysis of activation in an ROI in the anterior insula (left [−32 + 22 + 4]; right [+36 + 24 0]) did not show any significant group differences (effort: *t*(67) = 0.35, *p* = 0.726; reward: *t*(67) = 0.37, *p* = 0.710).

### fMRI: whole-brain analyses

Whole-brain analyses across both groups (one-sample t-tests) for the reward and effort modulators (decision/choice phase of trial) revealed, as expected, a network comprising the ACC, striatum, supplemental motor area, superior frontal gyrus, and parietal cortex (Table S9). Exploratory whole-brain two-sample *t*-tests comparing groups on reward- and effort-related brain activity found that the NMT group showed less activation to decisions with increasing effort levels in the left occipital cortex, compared to the MT group (peak voxel [−22 −74 + 22]; *t*(64) = 4.92, *k* = 341, *p* < 0.05 FWE cluster corrected).

### Correlations between brain activation, effort-based decision-making behavior, and psychological functioning at baseline

To shed light on the functional significance of the altered activation in the MT group in the ACC, correlational analyses were conducted separately in each group with regard to computational estimates for linear and quadratic effort sensitivity and measures of psychological functioning at baseline. In relation to the computational parameters, a positive correlation was observed in the MT group between ACC activation and effort sensitivity (linear: *r* = 0.439, *p* = 0.007; R^2^ = 0.19; quadratic: *r* = −0.437, *p* = 0.008; R^2^ = 0.19); these associations were non-significant in the NMT group (linear: *r* = −0.12, *p* = 0.512; R^2^ = 0.01; quadratic: *r* = −0.14, *p* = 0.423; R^2^ = 0.02). Fisher’s Z-tests showed that correlations with linear effort sensitivity differed significantly between groups (linear: *Z* = 2.35, *p* = 0.019; quadratic: *Z* =  1.3, *p* = 0.194). No significant correlations were detected between ACC activation and overall psychological functioning or internalizing symptoms at baseline (SI Table S8).

### Longitudinal results

**Fig. 3:**
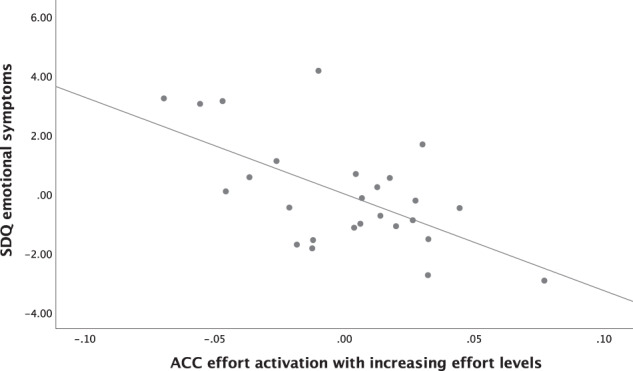
Partial regression plot. Prediction of internalizing symptoms from baseline ACC effort-related activation (with increasing effort levels) in the MT group, controlling for baseline emotional symptoms, sex, age, pubertal status, and MT severity.

In the MT group, ACC effort-related activation significantly predicted internalizing symptoms at follow-up (controlling for baseline emotional symptoms, sex, age, pubertal status and MT severity: ACC: *ß* = −0.41, *t*(18) = −2.98, *p* = 0.008; r^2^_part_ = 0.14; total model: *R*^*2*^ = 0.63, *F*(6,24) = 7.8, *p* < .001). A regression model including group as factor also showed a significant group by ACC activation interaction: *ß* = −0.26, *t*(48) = 2.06, *p* = 0.044; r^2^_part_ = 0.18 (total model: *R*^*2*^ = 0.61, *F*(6,55) = 12.8, *p* < 0.001). As shown in Fig. 3, participants with greater (i.e., more typical) baseline ACC effort-related activation had lower internalizing symptoms at follow-up.

### Sensitivity analyses

To probe our results’ robustness against extreme values, we conducted outlier analyses for the main findings. In all cases, the results remained unchanged, and in some cases were stronger after the removal of extreme values (SI; Table S11). However, as the populations under study are very heterogenous, we decided against the removal of those participants in our primary analyses.

## Discussion

The current study provides evidence that neural processing during a decision-making for reward task requiring manifest physical effort is altered in children with maltreatment experience, and predicts future internalizing symptoms. In line with our hypotheses, children with a history of childhood maltreatment relative to matched peers, presented with significantly lower effort-related activation in the ACC. Moreover, within the MT group a significant positive correlation between individual sensitivity to perceived effort and ACC activation was observed. Also, in line with our hypotheses, this attenuated effort-related activity in the ACC predicted increased internalizing symptoms in the MT group suggesting that altered ACC functioning during effort processing is implicated in a latent vulnerability mechanism conferring elevated risk of future psychopathology. By contrast, no group differences were found in the striatum in relation to reward or effort processing, nor were behavioral differences observed with regard to effort sensitivity.

Previous research has consistently implicated the ACC in effort-based decision-making [33, 36, 41]. More specifically, the dorsal part of the ACC has been proposed to act as an integrator of effort and reward information [41]; by contrast, it is not engaged when decisions only require a consideration of reward [43]. In our study, non-maltreated control participants on average showed a higher level of neural modulation in the ACC by perceived effort than the MT group. This is in line with previous findings indicating elevated ACC activation during effort processing in healthy adults [41]. The attenuated response in the ACC in the MT group is consistent with previous research showing that with increasing effort the ACC is less active in those with higher levels of apathy [36]. The ACC has been implicated in an array of other cognitive functions (see [45] for a review), including tracking aversiveness of tasks [56] and volatility of reward environments [57]. Further research with a focus on these processes is needed to refine our understanding of the role of the ACC and shed light on its potential role in the emergence of psychopathology after maltreatment. MT and NMT participants did not differ in how costly they perceived increasing levels of effort to be, as reflected in the computationally estimated measures of effort sensitivity. Interestingly, previous studies of children and adolescents with maltreatment experience also did not find performance differences on reward tasks [21] or a trend to quicker responses [7] or less modulation by reward [7, 22]. However, within the MT group, greater ACC activation was associated with greater effort sensitivity, reflecting more normative activation in children with higher levels of effort sensitivity.

Our longitudinal findings indicated that reduced ACC signaling in response to effort in children with maltreatment experience predicted higher levels of internalizing symptoms 18 months later. One possible interpretation is that in MT children attenuated ACC effort-related activity may represent a latent vulnerability marker of poorer emotional functioning. Previous research has reported a similar relationship between blunted reward response and future depressive symptomatology in adolescents with maltreatment experience [7, 27]. The current study extends these findings with regard to effort processing in the context of reward in children and adolescents with documented histories of maltreatment. This attenuation of ACC signaling in response to increasing effort may reflect the influence of atypical early environments characterized by maltreatment. Learning contingencies may be such that there are poorer signals to indicate when exerting effort might be worthwhile. We speculate that the observed attenuated ACC effort-related activity during decision making for reward may impact effortful control (the capacity to actively regulate behavioral and emotional responses) and reward-seeking behavior, both of which have been shown to be associated with future risk for internalizing symptoms [58, 59]. This suggestion is in line with the proposal that alterations in the reward and effort systems impair the ability of individuals to appropriately use affective information to make more optimal decisions and guide behavior [15]. Initially, this could present as diminished motivation to engage in pleasurable activities [33], reducing a child’s propensity to deploy effort when engaging in everyday challenging tasks at school or more broadly in social situations. However, over time, this could have a significant impact on internalizing problems, as well as psychosocial functioning, leading to less engagement in social interactions and activities as observed in adolescents at risk for depression [58]. This is also in line with research on animal models of early-life adversity showing increased anhedonia and changes in the hedonic-set point [29].

There were no significant group differences in the striatum (see Table S9 for information regarding group-level reward-related activation in this region). One possible explanation could be differences in the experimental paradigm and the relevant psychological subprocesses. While the task under study still involves a component of reward anticipation consistently associated with striatal functioning [28], previous research using the same effort-based decision-making reward paradigm has stressed the importance of frontal regions in these processes, especially the ACC, SMA, and premotor cortex [36]. Previous studies on the effects of MT on reward processing employed very different experimental designs, which do not involve an active acceptance or rejection of a trial by the participant [21, 25] or assessed the neural reactivity to pleasant stimuli not involving any explicit monetary rewards [8]. Future studies should investigate differences in subcomponents of reward processes after childhood maltreatment more systematically.

There are a number of limitations of this study that merit comment. First, we note that the sample size of the current study was constrained by the challenges of recruiting young people with a documented history of maltreatment experience in research, although this sample size is in line with several other studies in the field (e.g., [7, 60, 61]). Further research and replication are required. Although our power analyses for observed data indicated that our study was reasonably powered to detect group differences, previous research was typically based on relatively small samples which may have inflated effect sizes. Moreover, the relatively wide age range may have introduced some within-group heterogeneity (despite our matching on age and pubertal status) impacting our ability to detect group differences. As a consequence, we were not able to further analyze differences by sex or maltreatment type; moreover, there was a high degree of co-occurrence between maltreatment subtypes in line with the typical presentation of poly-victimization in this population [62]. Secondly, further follow-up data, including brain imaging data, would be highly desirable to characterize trajectories of behavioral and/or neural changes in reward and effort processing in more detail and help shed light on the functional significance of the observed neural alterations. Finally, within the current design, it is not possible to definitively attribute the observed neural changes to maltreatment experience; ethically it is not possible to assign participants to different conditions of childhood maltreatment status. However, given our careful matching of the groups and relevant evidence from the animal literature, we believe such a relationship is plausible. We also note that neural activation in the ACC did not correlate with our symptom measures at baseline, suggesting it was unlikely to simply reflect current pathology. It is possible that the neural differences observed between the groups reflect some form of a compensatory mechanism following maltreatment experience - future research is warranted to further explore this possibility.

The present results demonstrate that children who have experienced maltreatment are characterized by a pattern of altered neural processing during effort-based decision making for reward. We provide evidence that attenuated ACC effort-related activity for reward may represent a latent vulnerability marker for future internalizing symptoms. These findings highlight the importance of effort processing for reward as a potential target for preventative interventions following maltreatment experience.

## Supplementary information

Supplemental Material

Table S6

Table S9
