# Supplementary material for: Altered reward and effort processing in children with maltreatment experience: a potential indicator of mental health vulnerability
Source: Neuropsychopharmacology. 2022 Feb 11;47(5):1063–70. doi: 10.1038/s41386-022-01284-7 (PMC8832084; doi:10.1038/s41386-022-01284-7)
Supplement: Supplementary file 1 — Supplemental Material [file 41386_2022_1284_MOESM1_ESM.docx]

**Supplemental Information** for Altered reward and effort processing in children with maltreatment experience: A potential indicator of mental health vulnerability.

**Supplemental Methods**

|  | Maltreatment Group (N=39) | Non-maltreatment Group (N=37) | p |
| --- | --- | --- | --- |
| Sex, female: n (%) | 21 (54) | 21 (57) | .799 |
| Age *(SD)* | 13.7 *(2.0)* | 13.2 *(2.1)* | .369 |
| Pubertal status *(SD)* | 2.7 *(0.8)* | 2.3 *(0.9)* | .092 |
| Ethnicity, White: n (%) | 17 (44) | 16 (43) | .976 |
| Socioeconomic status *(SD)* | 3.3 *(1.0)* | 3.1 *(0.8)* | .434 |
| WASI-II IQ *(SD)* | 100.2 *(11.9)* | 102.9 *(9.6)* | .282 |

Table S1. Matching measures for full sample.

*Maltreatment history*

Fourteen (18%) randomly selected cases were double-rated by a senior social work professional. Interrater reliabilities showed 100% agreement in relation to the presence of sexual abuse, 92.8% for neglect and 85.7% for home violence and emotional abuse, respectively. NMT participant’s parents were asked whether they had ever been in touch with Social Services with regards to the child included in the study. In cases the answer was positive, it was clarified whether there had been any concerns about the child’s safety. If this was the case, the participant was excluded from the study.

|  |  | **N** | **Mean** | **SD** |
| --- | --- | --- | --- | --- |
| **Neglect** | Severity (0-4) | 24 | 2.06 | 1.72 |
|  | Duration |  | 5.73 | 4.18 |
|  | Age of onset |  | 5.42 | 3.51 |
| **Sexual abuse** | Severity (0-4) | 3 | 0.17 | 0.70 |
|  | Duration |  | 1.04 | 1.36 |
|  | Age of onset |  | 6.96 | 4.31 |
| **Emotional abuse** | Severity (0-4) | 33 | 2.11 | 1.13 |
|  | Duration |  | 6.73 | 5.13 |
|  | Age of onset |  | 8.03 | 2.12 |
| **Home violence** | Severity (0-4) | 30 | 1.31 | 1.24 |
|  | Duration |  | 4.29 | 4.03 |
|  | Age of onset |  | 5.48 | 2.69 |

Table S2. Documented maltreatment experience, severity, estimated duration and age of onset (both shown in years).

|  | Maltreatment Group (N=37) | Non-maltreatment Group (N=33) |
| --- | --- | --- |
| White British/White Irish/White Other, n [%] | 15 [41] | 13 [39] |
| Black British/Black African/Black Caribbean/Black Other, n [%] | 8 [22] | 9 [27] |
| Asian British/Asian Other, n [%] | 3 [8] | 2 [6] |
| Multiple ethnic groups, n [%] | 10 [27] | 7 [21] |
| Other, n [%] | 1 [3] | 2 [6] |

Table S3. Complete breakdown of ethnicities.

*Computational Analyses*

In addition to the standard model-agnostic analysis, we performed a computational analysis of accept/reject decisions in order to tease apart the contributions of different psychological processes on decision making. We were interested in comparing competing hypotheses of reward and effort contributions to decision making (model comparison and selection), as well as estimating model parameters corresponding to reward, effort, and other processes (parameter estimation). This allowed us to extract model parameters for each participant, compare groups, and examine associations with symptom measures.

All models were implemented in a hierarchical logistic regression framework, such that we could model trial-by-trial decisions based on the effort and reward on offer on a given trial. Hierarchical Bayesian estimation allowed us to recover parameter estimates more accurately than using typical maximum likelihood estimation. This is achieved through the application of soft constraints on likely parameter ranges using prior distributions [1]. Generatively, this modelling approach describes that participants are expected to come from a common group-level distribution, such that participants’ parameters are then expected to be similar to one another. Models were implemented using the probabilistic modelling language ‘Stan’ [2], and model parameters were estimated using Hamiltonian Markov Chain Monte Carlo (HMC) sampling. HMC sampling was chosen as it allows to estimate parameters both at the group level and individual subject level simultaneously.

A variety of models with increasing complexity were built to test and capture the contribution of reward and effort on decisions to engage or not on a given trial. We implemented 70+ models of varying complexity to test a variety of transformations of reward scaling and effort discounting onto subjective values of offers (see below). As a sanity check, we started testing simple assumptions such as participants performing randomly (Null model), and then iteratively improved the models until we could capture participants’ behavioural choice patterns with high fidelity. Once all models were fitted to the data, model comparison was performed to select the most parsimonious model (that is, the model that best captured the participants’ performance, whilst penalising for complexity). The Widely Applicable Information Criterion (WAIC) scale and K-fold cross validation (K-fold CV) were used to compare model fits [3, 4]. The WAIC is akin to cross-validation, or other approximations such as the Akaike Information Criterion, but the WAIC is more sensitive, particularly in hierarchical modelling settings [5]. K-fold CV was used when diagnostic measures of the WAIC (pareto-k) suggested that the WAIC approximation was sub-optimal. While K-fold CV provides true cross validation metrics as opposed to the approximation yielded by the WAIC, it requires refitting each model K times with different sub-partitions of the data, and as such is substantially more computationally expensive to compute than WAIC. A lower K-fold CV or WAIC for each model indicates a better model fit, and the relative difference between the winning model and another model can be used to establish the relative strength of the evidence for one model over another. This is akin to Bayes factors [6]. We defined a difference in model evidence (𝚫WAIC) of 0-2 as weak evidence; 2-6 positive evidence; 6-10: strong evidence; >10: very strong evidence in favour of the winning model [6].

For each model, two chains were produced with 1000 warm-up iterations and 4000 post warm-up iterations per chain. Model convergence was ensured through careful analysis of traceplots and monitoring of the Gelman-Rubin statistic (all potential scale reduction factors: R-hat < 1.1) [7, 8].

Recommendations for weakly informative priors in hierarchical logistic regression were used [1]. Following recent recommendations [9], prior predictive calibrations were performed on the winning model family to ensure that the prior choices led to adequate decision profiles. Parameter recovery on synthetic data was carried out to ensure that all parameters were identifiable and could be adequately recovered (Figure S1), and finally posterior predictive checks were performed to ascertain that the winning model could capture the behavioural choice pattern of all participants. For all models, subject-level individual parameters were normally distributed and drawn from the group level parameters (e.g. ; Figure S2a), with the exception of the noise term which was Beta distributed to enforce a proportion of noise level between zero and one:


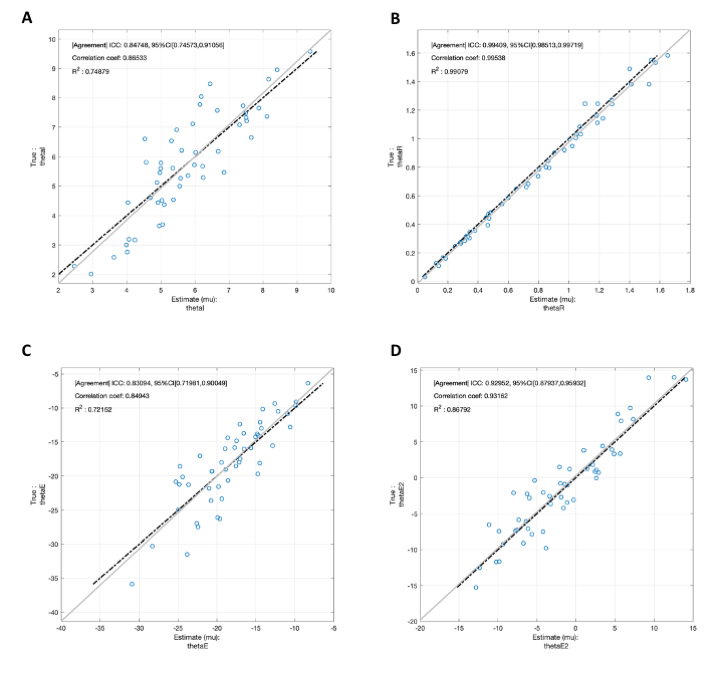
Figure S1. Parameter recovery from synthetic data: correlation between parameters used to create synthetic data and recovered parameters for A) intercept, B) reward, C) linear and D) quadratic effort.

*Winning Model.* The winning model is composed of five parameters per participant: two effort sensitivity parameters (linear and quadratic terms), a reward sensitivity term (linear), a bias term, and a noise term. Together all these parameters were able to capture the full range of choice patterns expressed by participants.

The model works as follows: On a given trial, the effort level (as indicated by the tree trunk bar) is transformed through the participant’s linear and quadratic effort sensitivity parameters to yield a subjective value of effort (equation S1). The linear effort sensitivity term scales the value of the effort level (the more negative, the more steeply true effort is discounted), and the quadratic effort sensitivity parameter allows the effort sensitivity profile to either taper off (Effort sensitivity^2^ > 0), or to increase disproportionately (Effort sensitivity^2^ < 0; Figure S2 c), as effort levels increase. The combination of the linear and quadratic effort sensitivity terms allows us to capture the range of effort sensitivity profiles observed in participants, which would not be possible with the linear or quadratic terms alone.

Similarly, on a given trial the reward level presented (number of apples) is transformed through each participant’s linear reward sensitivity term to yield a subjective value of reward (equation S2). This linear term scales the true magnitude of the rewards, such that if reward sensitivity equals one the rewards are taken at face value. If the reward sensitivity is lower than one, participants subjectively perceive rewards as being less rewarding than they ought to be, while a reward sensitivity > 1 results in perceiving rewards as more rewarding than they truly are (Figure S2b).

The subjective value of reward and effort are then linearly combined to form the subjective value of the offer (equation S3).

The subjective value of the offer is then passed through an invert logit link function with a bias parameter for each participant (equation S4). The invert logit function maps the subjective value of the offer to a probability of accepting the offer (Figure S2c), while the bias term enables to shift the subjective value by a constant. The bias term is equivalent to the baseline probability of accepting an offer, regardless of the reward and/or effort level present on the trial. The higher the bias term, the more likely a participant is to accept offers in general.

Finally, a proportion of random noise is allowed on each trial to account for and capture random performance on a subset of trials (e.g. due to inattention; equation S5). The higher the noise term, the more likely a participant will accept/refuse at random on a given trial, while a low noise term suggests a very deterministic participant.

*Alternative models and model comparison.* We tested a wide range of alternative models in an attempt to capture and compare the possible ways that participants could be subjectively transforming the true rewards and efforts presented on offer. We tested a variety of scalings for both effort and rewards: linear scaling (LinX), quadratic scaling (X^2^), exponential scaling (e^X^), combination of linear + quadratic scaling (LinX + X^2^), and finally tested the possibility of interactions between effort and rewards (RxE). We also tested the addition of both the impact of a noise term and intercept (baseline propensity to accept) on all these combinations of effort and reward scaling. This resulted in 70 different models testing for various combinations of strategies/mechanism that participants may use to decide whether to accept or refuse.

Of all the models implemented, a clear pattern emerged. Namely, a single family of model, with variations on a similar underlying structure, appears to best capture the performance of participants. The winning top 5 of the 70 models are as follows, in order of decreasing model evidence (best model first):

- Model 54: Intercept + Linear Reward + Linear Effort + Quadratic Effort + Noise
- Model 51: Intercept + Linear Reward + Linear Effort + ~~Quadratic Effort~~ + Noise
- Model 8: Intercept + Linear Reward + ~~Linear Effort~~ + Quadratic Effort + ~~Noise~~
- Model 9: Intercept + Linear Reward + Linear Effort + Quadratic Effort + ~~Noise~~
- Model 6: Intercept + Linear Reward + Linear Effort + ~~Quadratic Effort + Noise~~

The fact that the same effort and reward scaling approaches appear to win during model comparison (same family of models) for the top 5 models lends further credence to the winning model. This suggests that the strategy used by participants when subjectively transforming the true rewards and efforts on offer during the task appears to be a linear scaling of true rewards, and a linear plus quadratic scaling of effort.


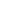

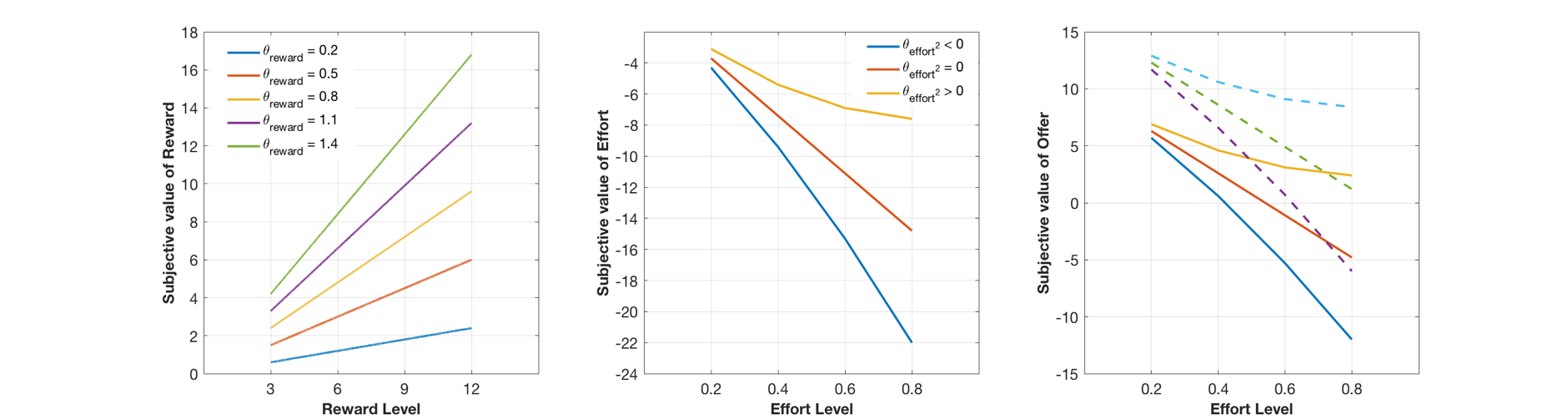

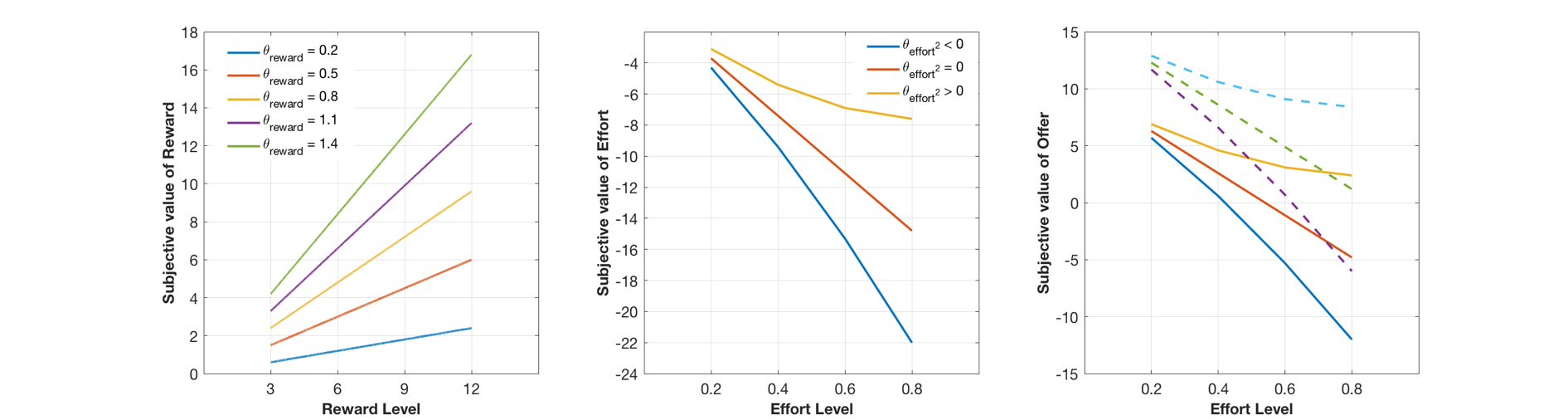

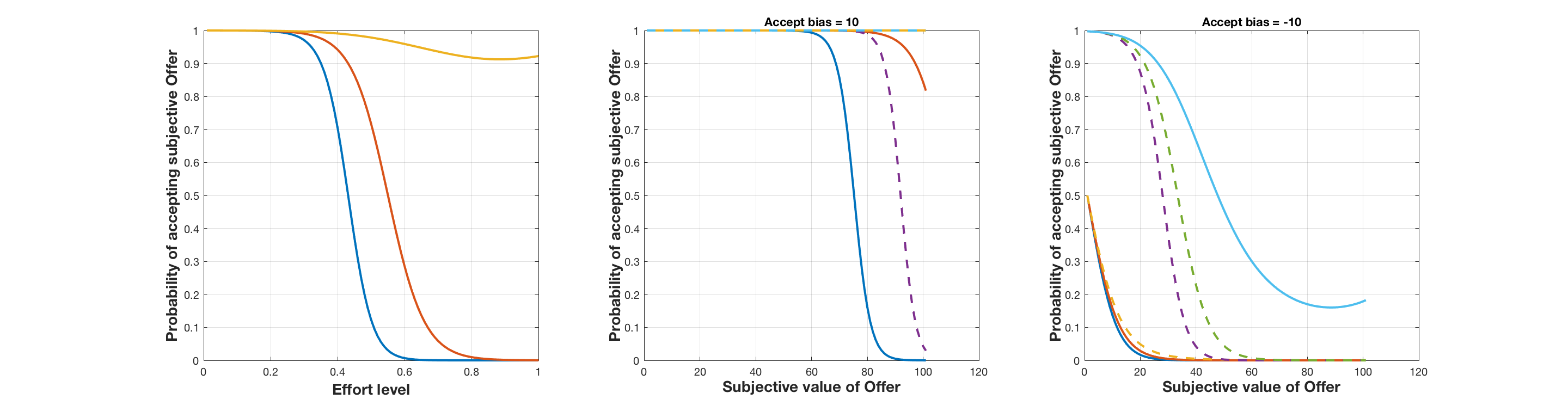


**a**

**b**

**c**

**d**

Figure S2. A) Graphical representation of the winning hierarchical model for the Apple Gathering Task. Participant-level parameters are drawn from a normal distribution, with the exception of the noise term which is Beta distributed. Group-level position and scale parameters are themselves drawn from normal and half-Cauchy distributions, respectively. B) Rewards on offer are scaled by the participants' reward sensitivity parameter, resulting in subjective valuations of the true reward magnitudes. C) The effort levels presented are scaled by the linear and quadratic effort sensitivity parameters, resulting in a subjective valuation of the true effort levels. D) A bias (intercept) term acts as a baseline propensity to accept offers, irrespective of the reward and effort levels present on the trial (baseline motivation).

*fMRI analyses*

*fMRI data acquisition*. Participants were scanned on a 1.5 Tesla Siemens Avanto MRI scanner (Siemens Medical Systems, Erlangen, Germany) using a 32-channel head coil and whole-brain multiband EPI sequence (multiband acceleration factor: 3, TR: 1300ms; TE: 54.3 milliseconds; voxel size: 3 x 3 x 3 mm; slices per volume, 39; slice thickness: 3mm; field of view: 192 mm; flip angle: 62 degree). Participants completed three task runs lasting approximately 6 min resulting in 210 volumes on average. A magnetization-prepared rapid gradient-echo sequence (MP-Rage) was used to obtain a high-resolution structural scan (parameters: 176 slices; slice thickness: 1 mm; gap between slices: 0.5 mm; TR: 2730 milliseconds; TE: 3.57 milliseconds; voxel size: 1 x 1 x 1 mm; field of view: 256 mm).

*FMRI preprocessing*. After discarding the first eight volumes to allow for magnetic equilibration, volumes were realigned to the first image and unwarped using SPM12’s default options. Next, the anatomical scan was co-registered to the mean functional image. Using the deformation parameters of the anatomical scan’s segmentation at a 3 x 3 x 3 voxel size, functional images were normalized into Montreal Neurological Institute (MNI) standard space, smoothed using an 8 mm Gaussian kernel.

Frame-to-frame displacement was assessed and images displaying corruption following large movements (> 1.5 mm or > 0.5 degree rotation) were replaced by interpolation of unaffected adjacent images. Groups did not differ significantly on average absolute movement (MT: mean=.12, SD=.06; NMT: mean=.13, SD=.08; *t*(66)=.73, *p*=.471).

*Subject-level analysis.* General linear models included an autoregressive term, a high pass filter at 128 Hz as well as six motion regressors to reduce motion-related artifacts. Furthermore, one regressor for each scan that was interpolated due to corruption was included. To account for possible effects of physiological artifacts, timeseries from white matter and CSF regions were also included. These were extracted using the TAPAS toolbox implemented in CONN [10; www.nitrc.org/projects/conn, RRID:SCR_009550] using the first five principal components at a threshold of 0.9 [11, 12].

*Second-level analysis.* Whole brain results were analysed applying a stringent correction for multiple comparisons using AFNI’s latest 3dttest++ in combination with the 3dClustSim routine (‘-Clustsim’ option) (<https://afni.nimh.nih.gov/pub/dist/doc/program_help/3dClustSim.html>), which uses permutation testing to derive cluster size thresholds corresponding to p < .05 FWE corrected.

**Supplemental Results**

*Effects of Covid-19 pandemic*

Until the first lockdown in England due to the Covid-19 pandemic 6 MT participants out of a total of 27 and 5 NMT participants out of a total of 31 had completed the follow-up. After a transition period, we started to collect data on the potential impact of the pandemic on participants’ emotional well-being. Initially the following two questions were asked: ‘How significant has the impact of COVID-19 been on your family on a scale of 1 to 10?’ and ‘How much has your child’s emotional well-being been impacted by COVID-19 on a scale of 1 to 10?’. After obtaining the respective ethics committee’s decision we used the Coronavirus Health Impact Survey (CRISIS) ‘Baseline Current Form’ (<https://github.com/nimh-comppsych/CRISIS>) and an emotional impact subscale was calculated from items 3, 4, 5, 6, 16, 18, 19, 21 and 22 which showed good internal consistency (Cronbach’s alpha = 0.84). Results showed that according to these measures at the time of follow-up, the emotional impact of the pandemic had been similar for MT and NMT participants (Table S4).

|  | MT  Mean (SD) | NMT  Mean (SD) | p |
| --- | --- | --- | --- |
| Follow-up interval (days) *(SD)* | 578.4 *(36.0)* [N=27] | 572.4 *(28.3)*  [N=31] | .475 |
| ‘How significant has the impact of COVID-19 been on your family on a scale of 1 to 10?’ | 5.3 (2.7)  [N=15] | 5.2 (3.2)  [N=25] | .924 |
| ‘How much has your child’s emotional well-being been impacted by COVID-19 on a scale of 1 to 10?’ | 4.4 (2.6)  [N=15] | 4.6 (3.7)  [N=25] | .972 |
| Subscale from CRISIS ‘Emotional Impact of Pandemic’ | 16.6 (9.1)  [N=11] | 15.2 (5.0)  [N=22] | .629 |
| Number of participants reporting negative life-events in Coddington | 3 | 1 | .135 |

Table S4. Descriptive Statistics and group comparisons for follow-up data.

*Analysis of Behavior*

Descriptive statistics can be found in Table S5. Figure S3 A) shows mean success rates by reward and effort level, Figure S3 B) shows mean response times by reward and effort level. Three separate ANOVAs were conducted in SPSS 25 for acceptance rates, success rates and response times (RTs) as dependent variables, with within-subjects factors of reward (3 levels) and effort (3 levels), and the between-subjects factor group. As the assumption of sphericity was violated, the values according to the Greenhouse-Geisser correction are reported in Table S6.

| **Descriptive Statistics** | | | | |
| --- | --- | --- | --- | --- |
|  | MT  Mean SD | | NMT  Mean SD | |
| Acceptance Rate [%] | 86.3 | 9.5 | 80.6 | 8.4 |
| Success Rate [%] | 67.3 | 11.0 | 65.8 | 10.9 |
| RTs [ms] | 984.2 | 176.7 | 1032.2 | 239.4 |
| Total Reward [points collected] | 344.2 | 70.6 | 332.6 | 69.5 |

Table S5. Descriptive statistics for acceptance rate, success rate, RT, total reward.


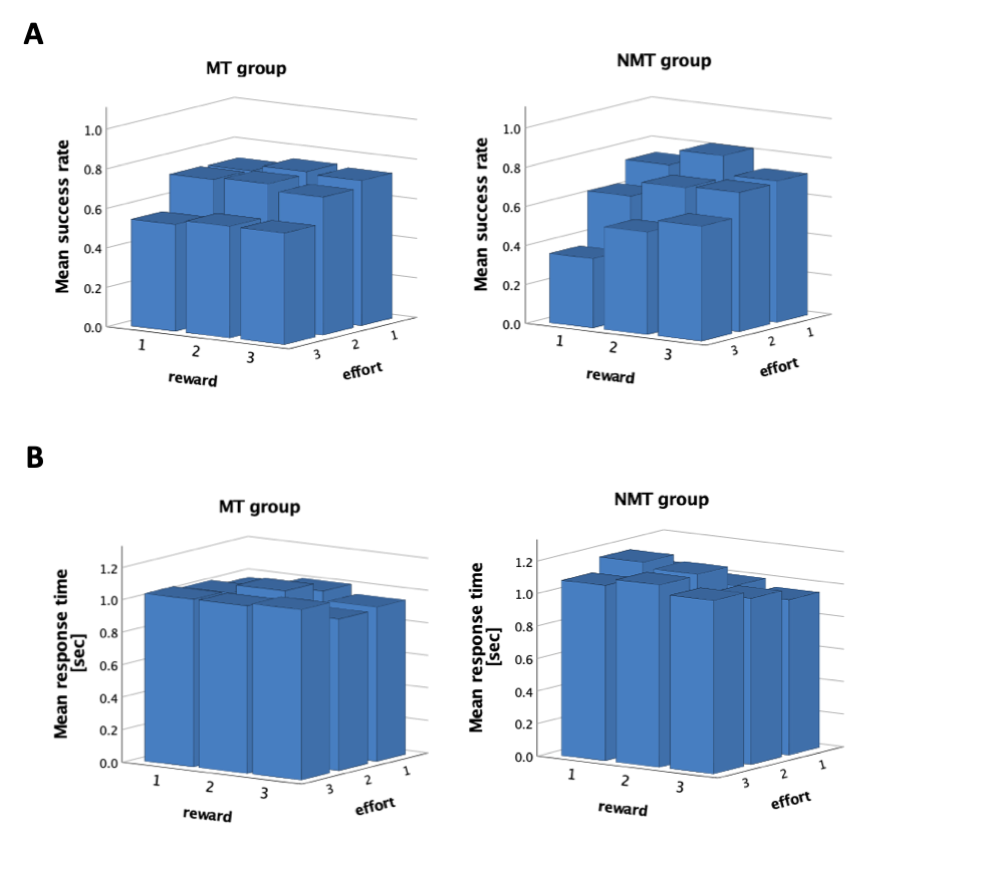


Figure S3. A) Mean success rates by reward and effort level for MT and NMT group. B) Mean response times by reward and effort level for MT and NMT group.

| **ANOVA on acceptance rates** | | | |
| --- | --- | --- | --- |
| Effect | F | df | p |
| Main effect reward | 53.4 | 1.32, 89.5 | < .001^*^ |
| Main effect effort | 90.2 | 1.23, 83.7 | < .001^*^ |
| Main effect group | 4.5 | 1, 68 | .037 |
| Interaction reward * effort | 26.5 | 2.60, 176.6 | < .001^*^ |
| Interaction reward * group | 1.5 | 1.32, 157.5 | .221 |
| Interaction effort * group | 1.9 | 1.23, 151.7 | .163 |
| Interaction reward * effort * group | 0.8 | 2.60, 244.6 | .437 |
| **ANOVA on success rates** | | | |
| Effect | F | df | p |
| Main effect reward | 5.3 | 1.90, 89.2 | .008^*^ |
| Main effect effort | 28.6 | 1.60, 75.3 | < .001^*^ |
| Main effect group | 0.5 | 1, 47 | .480 |
| Interaction reward * effort | 1.6 | 3.42, 160.6 | .175 |
| Interaction reward * group | 2.3 | 1.90, 136.2 | .114 |
| Interaction effort * group | 1.0 | 1.6, 122.3 | .356 |
| Interaction reward * effort * group | 2.1 | 3.4, 207.6 | .094 |
| **ANOVA on RTs** | | | |
| Effect | F | df | p |
| Main effect reward | 1.6 | 1.59, 74.5 | .208 |
| Main effect effort | 2.8 | 1.68, 79.1 | .078 |
| Main effect group | 0.6 | 1, 47 | .426 |
| Interaction reward * effort | 2.9 | 2.95, 138.5 | .037 |
| Interaction reward * group | 0.6 | 1.59, 121.5 | .521 |
| Interaction effort * group | 0.3 | 1.68, 126.1 | .732 |
| Interaction reward * effort * group | 0.6 | 2.95, 185.5 | .600 |

Table S6. ANOVA results for behavioral indices acceptance rate, success rate, RT.

* p < .05 surviving correction for multiple comparison.

| **MT group** | | | |
| --- | --- | --- | --- |
|  |  | SDQ Total | SDQ emotional |
| Reward Sensitivity | r | -.38 | -.19 |
|  | p | .023 | .261 |
| Effort Sensitivity | r | -.15 | -.17 |
|  | p | .391 | .327 |
| **NMT group** | | | |
|  |  | SDQ Total | SDQ emotional |
| Reward Sensitivity | r | -.07 | -.24 |
|  | p | .720 | .171 |
| Effort Sensitivity | r | .18 | .20 |
|  | p | .324 | .261 |

Table S7. Correlation analyses between computationally derived reward / effort sensitivity and symptom score separately for groups.

| **MT group** | | |
| --- | --- | --- |
|  |  | ACC effort-related activation |
| Linear Effort Sensitivity | r | .44* |
|  | p | .007 |
| Quadratic Effort Sensitivity | r | .44* |
|  | p | .008 |
| SDQ Total score | r | -.20 |
|  | p | .233 |
| SDQ emotional | r | -.11 |
|  | p | .534 |
| **NMT group** | | |
| Linear Effort Sensitivity | r | -.12 |
|  | p | .512 |
| Quadratic Effort Sensitivity | r | -.14 |
|  | p | .423 |
| SDQ Total score | r | -.13 |
|  | p | .479 |
| SDQ emotional | r | .02 |
|  | p | .909 |

Table S8. Correlation analyses between ACC effort-related activation and computationally derived reward / effort sensitivity and symptom scores at baseline separately for groups.

* p < .05 surviving correction for multiple comparison.

| **Neural activation with increasing reward** | | | | | |
| --- | --- | --- | --- | --- | --- |
|  | Coordinates  x y z | | | T | k |
| ACC* | +10 | +6 | +50 | 4.69 | 182 |
| Left striatum | -8 | +4 | 0 | 5.98 | 43 |
| Right insula / inferior frontal gyrus* | +36 | +22 | +10 | 4.44 | 124 |
| Left medial frontal gyrus* | -22 | -18 | +62 | 4.18 | 225 |
| Superior parietal cortex* | -26 | -62 | +64 | 3.79 | 65 |
| **Neural activation with decreasing reward** | | | | | |
| Medial occipital cortex* | -16 | -68 | +12 | 5.08 | 554 |
| Left postcentral gyrus* | -62 | -12 | +14 | 5.05 | 87 |
| Left superior temporal cortex* | -56 | -28 | +2 | 4.47 | 65 |
| Right parahippocampal gyrus | +24 | -30 | -16 | 4.76 | 41 |
| **Neural activation with increasing effort** | | | | | |
| ACC* | 0 | +32 | +18 | 6.23 | 198 |
| Middle cingulate cortex | +2 | -18 | +32 | 4.81 | 46 |
| Precentral gyrus / supplemental motor area / superior parietal cortex* | +22 | -32 | +68 | 6.12 | 2122 |
| Superior frontal gyrus | +2 | +22 | +54 | 3.89 | 41 |
| Left precentral gyrus | -46 | -14 | +36 | 5.44 | 92 |
| Right inferior parietal cortex* | +48 | -52 | +38 | 4.16 | 198 |
| Right fusiform gyrus* | +38 | -76 | -14 | 4.69 | 164 |
| Midbrain / thalamus* | 0 | -36 | +6 | 5.63 | 131 |
| Midbrain / substantia nigra | +2 | -20 | -12 | 5.12 | 98 |
| **Neural activation with decreasing effort** | | | | | |
| Right superior parietal cortex* | +16 | -64 | +60 | 6.79 | 2122 |
| Left superior parietal cortex* | -18 | -62 | +60 | 6.75 | 2138 |
| Left middle frontal gyrus* | -22 | 0 | +58 | 6.03 | 575 |
| Right inferior frontal gyrus* | +60 | +8 | +16 | 5.38 | 240 |
| Left insula* | -50 | +8 | +2 | 4.98 | 195 |

Table S9. Whole-brain parametric activations with increasing and decreasing individual reward / effort sensitivity respectively using computationally derived parameters, across groups during decision/choice phase; for illustrative purposes thresholded at p < 0.001, k = 40 (uncorrected).

* survive FWE cluster-level correction

*Analysis of fMRI data*

In order to check the robustness of the major fMRI ROI and correlational findings, we additionally analysed models with actual reward and effort levels as parametric modulators instead of computationally derived parameters. Overall, result patterns were similar but as expected these analyses were less sensitive to detect group differences as shown in Table S10.

| Regions of Interest | | | | | | | |
| --- | --- | --- | --- | --- | --- | --- | --- |
|  | | | | Comparison for reward-related activity | | Comparison for effort-related activity | |
|  | Coordinates  x y z | | | T | p | T | p |
| ACC | 0 | +14 | +46 | 0.74 | .465 | 1.74 | .086 |
| Right striatum | +22 | +14 | +6 | -0.07 | .942 | -0.47 | .645 |
| Left striatum | -14 | +6 | +10 |

Table S10. ROI results with actual reward and effort level as parametric modulators.

*Correlation of effort sensitivity and ACC activity based on actual effort level*

Similar to the results based on the computationally derived parameters, there was a positive correlation between ACC effort-related activity and individual linear effort sensitivity in the MT group (r=.39, *p*=.019; *R^2^*=.152) whereas this correlation was non-significant in the NMT group (*r*=-.04, *p*=.838; *R^2^*=.001). Fisher’s Z-test narrowly missed significance (*Z*=1.78, *p*=.075).

*Sensitivity analyses.* For normally distributed measures Tukey’s box-plot Interquartile Range (IQR) method was used to identify outliers whereas for non-normally distributed measures the adjusted boxplot method developed by Hubert and Vandervieren [13] was implemented. For both methods a 1.5 interquartile range (IQR) was applied. Multivariate analyses were assessed using studentized regression residuals [14]. Results can be found in Table S11.

| **Measure** | **Statistical value including all** | **p value including all** | **Statistical value after exclusion** | **p value after exclusion** | **Number of identified extreme values in  MT group** | **Number of identified extreme values in NMT group** |
| --- | --- | --- | --- | --- | --- | --- |
| ACC effort activation (non-normal) | *t*(67) = -2.19 | .032 | *t*(60.23) =  -2.6 | .010 | 0 | 2 |
| ACC effort activation – linear effort sensitivity (MT group) | r = .44 | .007 | - | - | 0 | - |
| ACC effort activation – linear effort sensitivity (NMT group) | r = -.12 | .512 | r = -.14 | .464 | - | 2 |
| ACC effort activation – quadratic effort sensitivity  (MT group) | r = .44 | .008 | - | - | 0 | - |
| ACC effort activation – quadratic effort sensitivity  (NMT group) | r = -.14 | .423 | r = -.20 | .276 | - | 2 |

Table S11. Results of outlier analyses for major findings.

**Supplemental Reference List**

1. Gelman, A. and J. Hill, *Data Analysis Using Regression and Multilevel/Hierarchical Models.* 2007: Cambridge University Press.

2. Carpenter, B., et al., *Stan: A Probabilistic Programming Language.* Journal of Statistical Software, 2017. **76**(1): p. 1-29.

3. Watanabe, S., *A Widely Applicable Bayesian Information Criterion.* Journal of Machine Learning Research, 2013. **14**: p. 867-897.

4. Vehtari, A., A. Gelman, and J. Gabry, *Practical Bayesian model evaluation using leave-one-out cross-validation and WAIC.* Statistics and Computing, 2017. **27**(5): p. 1413-1432.

5. Gelman, A., J. Hwang, and A. Vehtari, *Understanding predictive information criteria for Bayesian models.* 2013.

6. Kass, R.E. and A.E. Raftery, *Bayes Factors.* Journal of the American Statistical Association, 2012. **90**(430): p. 773-795.

7. Brooks, S.P. and A. Gelman, *General Methods for Monitoring Convergence of Iterative Simulations.* Journal of Computational and Graphical Statistics, 2012. **7**(4): p. 434-455.

8. Gelman, A. and D.B. Rubin, *Inference from Iterative Simulation Using Multiple Sequences.* Statistical Science, 1992. **7**(4): p. 457-472.

9. Gabry, J., et al., *Visualization in Bayesian workflow.* , in *arXiv.org*. 2017, September 5, John Wiley & Sons, Ltd.

10. Whitfield-Gabrieli, S. and A. Nieto-Castanon, *Conn: a functional connectivity toolbox for correlated and anticorrelated brain networks.* Brain Connect, 2012. **2**(3): p. 125-41.

11. Behzadi, Y., et al., *A component based noise correction method (CompCor) for BOLD and perfusion based fMRI.* Neuroimage, 2007. **37**(1): p. 90-101.

12. Li, Y., R. Saxe, and S. Anzellotti, *Intersubject MVPD: Empirical comparison of fMRI denoising methods for connectivity analysis.* PLoS One, 2019. **14**(9): p. e0222914.

13. Hubert, M. and E. Vandervieren, *An adjusted boxplot for skewed distributions.* Computational Statistics & Data Analysis, 2008. **52**(12): p. 5186-5201.

14. Stevens, J.P., *Outliers and Influential Data Points in Regression-Analysis.* Psychological Bulletin, 1984. **95**(2): p. 334-344.
